# Regulation of Pancreatic Beta Cell Stimulus-Secretion Coupling by microRNAs

**DOI:** 10.3390/genes5041018

**Published:** 2014-11-06

**Authors:** Jonathan L. S. Esguerra, Inês G. Mollet, Vishal A. Salunkhe, Anna Wendt, Lena Eliasson

**Affiliations:** Unit of Islet Cell Exocytosis, Department of Clinical Sciences in Malmö, Lund University Diabetes Centre, Lund University, CRC 91-11, Jan Waldenströms gata 35, 205 02 Malmö, Sweden; E-Mails: jonathan.esguerra@med.lu.se (J.L.S.E.); ines.mollet@med.lu.se (I.G.M.); vishal-salunkhe@med.lu.se (V.A.S.); anna.wendt@med.lu.se (A.W.)

**Keywords:** MicroRNA, miRNA, non-coding RNA, islet, beta cell, insulin, glucose, insulin secretion, exocytosis, SNARE

## Abstract

Increased blood glucose after a meal is countered by the subsequent increased release of the hypoglycemic hormone insulin from the pancreatic beta cells. The cascade of molecular events encompassing the initial sensing and transport of glucose into the beta cell, culminating with the exocytosis of the insulin large dense core granules (LDCVs) is termed “stimulus-secretion coupling.” Impairment in any of the relevant processes leads to insufficient insulin release, which contributes to the development of type 2 diabetes (T2D). The fate of the beta cell, when exposed to environmental triggers of the disease, is determined by the possibility to adapt to the new situation by regulation of gene expression. As established factors of post-transcriptional regulation, microRNAs (miRNAs) are well-recognized mediators of beta cell plasticity and adaptation. Here, we put focus on the importance of comprehending the transcriptional regulation of miRNAs, and how miRNAs are implicated in stimulus-secretion coupling, specifically those influencing the late stages of insulin secretion. We suggest that efficient beta cell adaptation requires an optimal balance between transcriptional regulation of miRNAs themselves, and miRNA-dependent gene regulation. The increased knowledge of the beta cell transcriptional network inclusive of non-coding RNAs such as miRNAs is essential in identifying novel targets for the treatment of T2D.

## 1. Introduction

Deregulated miRNA expression is thought to be implicated in the pathogenesis of many diseases [1], including metabolic disorders such as type 2 diabetes (T2D) [2]. Years of genetic, cell biological and physiological research, since the discovery of the first miRNA, lin-4 [3], have contributed to our knowledge concerning the importance of miRNAs in the control of diverse cellular processes. MiRNAs are single-stranded, short (20–25 nt) non-coding RNAs that mediate post-transcriptional gene silencing of target genes through translational inhibition, mRNA degradation and/or deadenylation. Silencing happens through the sequence complementarity of the so-called 7–8 nt long “seed sequence” with the target mRNA sequence usually in the 3'UTR to guide the RNA-induced silencing complex (RISC), a ribonucleoprotein complex, to target the mRNAs. The canonical “seed sequence” is a prerequisite for target recognition. The multidomain protein argonaute is part of RISC and has a central role in the repression of the target gene [4]. In plants, ~100% complementarity between mature miRNA and mRNA target site is required. In mammals, partial base-pairing is also feasible, thereby allowing one miRNA to influence the expression of several targets, and several miRNAs to regulate the expression of a single gene [5]. Hence, this complex network organization has made miRNAs essential in many cellular processes [6], from cell development to the control of physiological processes such as insulin secretion [7,8].

Insulin is secreted by the endocrine pancreatic beta cells upon an increase in blood glucose to lower the blood glucose level. The increased blood glucose, usually occurring after a meal (postprandial), is sensed by the beta cell and glucose is taken up by glucose transporters. Once inside, glucose is first converted to glucose-6-phosphate by glucokinase and then in the glycolysis to pyruvate, which enters the mitochondria [9]. One important feature of the beta cell is the low levels of MCT-1, which prevents exogenous pyruvate to enter and insulin to be released at low glucose during exercise [10]. Metabolism of glucose yields an increased ATP-level which contributes to closure of the ATP-dependent K^+^ channel. This channel consists of an inward rectifier channel (KCNJ11) and the sulfonylurea receptor (ABCC8) [11], and is responsible for maintaining the membrane potential of the beta cell hyperpolarized at low glucose [9]. Once closed, the membrane potential is depolarized, and voltage-dependent Ca^2+^ channels are opened. The subsequent influx of Ca^2+^ triggers exocytosis of insulin granules and the release of insulin [12]. A plethora of Ca^2+^ channels are involved in this process. Recent studies have pointed out large differences between mouse and human beta cells regarding different channel sub-types involved [9]. These species’ differences might be explained by species-specific non-coding RNA gene expression regulation [13]. For instance, we recently detected several long non-coding RNAs associated with HbA1c in human pancreatic islets, which are not conserved in rodents [14].

The exocytotic machinery in the pancreatic beta cells involves several proteins [15]. These include the SNARE-proteins, SNAP-25, syntaxin 1 [16,17,18], VAMP-2 (or synaptobrevin) [19], several synaptotagmins [20,21], and other proteins such as granuphilin (also known as synaptotagmin-like protein 4a; slp4a) and munc-18 (or stxbp1) [16,20,22]. Prior to Ca^2+^-dependent fusion of the large dense core vesicles (LDCVs) with the plasma membrane, the LDCVs need to go through a process called priming to become ready for release. Priming is an ATP-, Ca^2+^- and temperature-dependent process that can be amplified in the presence of cAMP [23]. The latter involves the activation of the cAMP-dependent protein Epac2 [24,25], ClC3 chloride channels [26] and CFTR [27].

Failure of the beta cell to release enough insulin leads to increased blood glucose levels. Indeed, one of the hallmarks of T2D is impaired insulin secretion. Our knowledge of T2D pathogenesis has increased during the recent years and many of the common genetic variations associated with the disease have been linked to pancreatic beta cell functions [28]. However, genetic variations only explain a small part of the increased risk and other factors beyond the genome have been suggested to be involved, e.g., epigenetic regulation and miRNAs. It is obvious from recent studies that epigenetic marks and miRNAs play crucial roles in the regulation of human insulin secretion [29,30,31,32,33]. It is of interest that expression of many exocytotic genes is downregulated in islets from T2D donors [20,34] and yet a very limited number of common variations in or nearby these genes have been linked to their expression [20].

In this review, we will focus on the role of miRNAs in beta cell stimulus-secretion coupling and exocytosis. We will discuss (1) regulation of miRNAs by glucose and transcription factors, and (2) how essential components of the stimulus-secretion coupling are controlled by miRNAs. The balance between these two processes is essential for healthy beta cells; increased understanding of the interplay between them will most likely improve our possibilities of identifying new therapeutic approaches against beta cell impairment in T2D.

## 2. Transcriptional Regulation of miRNAs in Beta Cells

If miRNAs are to have a major function in the regulation of the different components of beta cell stimulus-secretion coupling, it is likely that the expression of these components are regulated by similar agents, such as glucose and the incretin hormone GLP-1. Indeed, several islet miRNAs are glucose regulated [35,36,37]. Exactly how this regulation is effected remains largely to be determined.

Important effects of incretins on islets are mediated through cAMP, one being transcriptional regulation of genes having central roles in insulin secretion, such as the insulin gene [38]. Cyclic-AMP regulates the expression by activation of CREB that bind to CRE sites at the promoter region. Most likely, this mechanism of regulation is also valid for miRNA expression through regulation of CRE sites on miRNA promoters. Indeed, we have preliminary data that shows cAMP-dependent regulation of miR-212 and miR-132 [39].

Transcriptional regulation of primary miRNAs (pri-miRNAs) has not been extensively studied. However, in spite of the fact that most metazoan miRNA genes do not appear to have the classical signals for polyadenylation [40], there is evidence suggesting that most miRNAs have the same type of promoters as protein-coding genes, including proximal promoter sequences, and distal upstream and/or downstream enhancers. Most miRNAs appear to be transcribed by RNA polymerase II [5,41] and are therefore capped. Some miRNAs, nonetheless, can be transcribed by RNA polymerase III [5,42]. As far as we know there are no transcription factors specific to only miRNAs. On the other hand, many miRNAs are located within exons or introns of functional spliced genes, including both protein-coding genes and non-coding genes. Although some may be regulated through the promoter of the host gene, evidence from Ozsolak *et al.* [43] suggests that one-third of intronic miRNAs possess transcriptional control regions which may function independently of their host gene promoters both with RNA polymerase II- and III-occupied miRNAs. In addition, miRNAs can be clustered into polycistronic intergenic transcripts and there is evidence that these are coordinately regulated [44]. For example, in islets from the diabetic GK rats, several differentially expressed miRNAs [35] are found clustered on the genome: e.g., (rno-miR-212/rno-miR-132), (rno-mir-376c/rno-mir-376b/rno-mir-376a), and (rno-mir-409/rno-mir-369/rno-mir-410).

Other regulators than transcription factors have also been shown to modulate miRNA expression, e.g., it has been demonstrated that thioredoxin-interacting protein TXNIP, which is upregulated by glucose in the diabetic state, downregulates miR-124a expression [45]. This miRNA is known to directly target forkhead box A2 (FoxA2) [45,46] a transcription factor which in turn targets islet amyloid polypeptide (IAPP) [45] and the KiR6.2 and SUR1, components of the ATP-dependent K^+^ channel [46].

Several miRNAs appear to be cell-specific or cell-enriched, *i.e.* more abundant in a certain cell type, as it is the case for miR-375 in beta cells. This suggests that the defined profile of transcription factors that maintains cell specificity may also regulate the expression of such miRNAs. Avnit-Sagi *et al.* [47] have identified several conserved regions on the miR-375 promoter and E box elements, which suggests that this miRNA may be regulated by basic-Helix-Loop-Helix (bHLH) transcription factors such as Ngn3 and NeuroD1. The latter two are central for beta cell maturation and maintenance. Moreover, in the knockout model of miR-375, the ratio of alpha to beta cells is severely disturbed, resulting in hyperglycemia [48]. This is also observed in beta cell-specific knockout models of the enzyme Dicer [37,49,50], thus demonstrating the importance of miRNAs in the development and maturation of the pancreatic islet cells. Indeed, specific miRNAs are differentially expressed during the period of perinatal beta-cell expansion and maturation in rats [51].

Interestingly, some miRNAs present in beta cells essentially target transcriptional regulation, as is the case for miR-212 and miR-132. Here we see the addition of complex feedback control mechanisms by miRNAs on the transcription factor pool itself. This example lends substance to the view that miRNAs may function as buffers preventing random environmental cues from resulting in signaling that might lead the system astray. One example of such a feedback control involves the transcriptional repressor methyl CpG-binding protein 2 (MeCP2), which is both a target and a transcriptional regulator of miR-212 [52]. Interestingly, MeCP2 is also involved in the regulation in beta cells of Arx (the Aristaless homeobox gene) a central player implicated in maintenance of beta cell identity [53].

**Figure 1:**
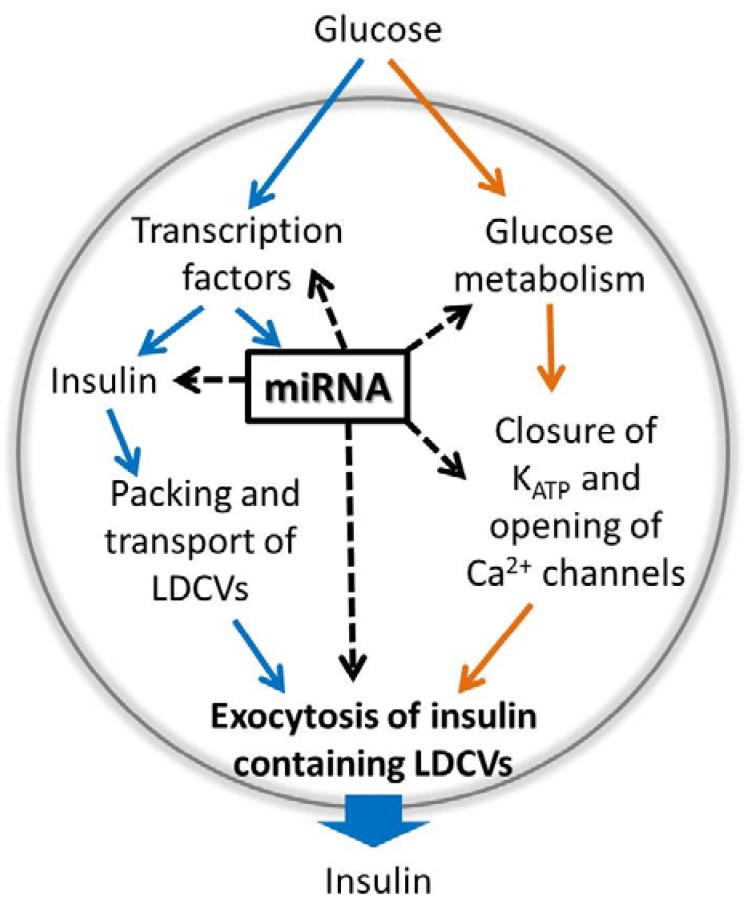
A model figure describing glucose-induced expression of the insulin gene and miRNAs on the left side (blue arrows) and glucose-regulated insulin exocytosis and secretion on the right side (orange arrows). As indicated by the dotted black arrows, miRNAs regulate gene expression of several proteins involved in these processes as listed in Table 1. A majority of miRNA targets is discovered among the exocytotic proteins. Notice the possible feedback regulation involving transcription factors and miRNAs.

The future will tell more about the complex regulation of miRNAs and the feedback loops that might be involved. Evidently part of the miRNA pool is regulated by glucose and/or cAMP, although the exact pathways involved are yet to be revealed (Figure 1).

## 3. Control of Beta Cell Stimulus-Secretion Coupling by miRNAs

The route from glucose uptake to insulin release is fairly rapid and occurs in the time frame of minutes. The first phase insulin secretion is normally 5–15 min long and involves several thousand action potentials, each lasting ~50 ms [9]. As for the generation of action potentials the process of exocytosis is a rapid process. Hence, any putative miRNA-dependent regulation of genes during the stimulus-secretion coupling also needs to be very fast. Although a rapid miRNA-mediated gene regulation has not yet been shown in the context of glucose-stimulated insulin secretion in the beta cell, such a scenario is possible as exemplified by experiments performed in retinal neurons [54]. However, examples of slower miRNA-induced expression changes are available and even more common. These are often due to long-term adaptations such as development of a disease state such as diabetes [2]. Below we exemplify genes in the stimulus-secretion coupling controlled by miRNAs (Table 1 and Figure 1).

### 3.1. “Fuel-Uptake and Glucose Metabolism”-Related Genes Controlled by miRNAs

Increased glucose concentration in the blood leads to its uptake into beta cells via glucose transporters, where the major isoforms are GLUT-1 and GLUT-3 in human, and glut2 in rodent beta cells [55]. Glucose is converted to glucose-6-phospahte by the enzyme glucokinase followed by mitochondrial metabolization and production of ATP. The 3'UTR of glucose transporter transcripts is predicted to harbor miRNA-binding sites, though this has not been experimentally validated for any beta cell glucose transporters. In the gall bladder carcinoma cell line T24, the expression of *glut-3* has been demonstrated to be regulated by miR-195-5p [56].

The beta cell has evolved highly specialized functions to maintain glucose homeostasis at the cost of strict regulation of some genes. For instance, during exercise, pyruvate and lactate produced in the muscle, which would otherwise stimulate insulin secretion, are prevented from being metabolized in the beta cell. The high selectivity for glucose-derived pyruvate as metabolic intermediate in the beta cell is achieved by both the low expression of lactate dehydrogenase (LDHA) [57] and reduced level of monocarboxylate transporter (MCT11; SLC16A1) [58]. The latter is suppressed by a number of miRNAs, such as miR-29a/b and miR-124 [59]. This exemplifies a situation when high miRNA expression is favorable, aimed at constitutive repression of so-called “disallowed genes” in the beta cell [10,60,61]. 

**Table 1:** miRNAs implicated in regulating components of glucose-stimulated insulin secretion.

GSIS Process	miRNA–mRNA Interactions ^1^	Model System ^2^	Ref.
Glucose or fuel uptake and glucose metabolism	miR-29 a/b, miR-124 --| *Mct1*	MIN6	[59]
	miR-195-5p --| *Glut3*	T24	[56]
	miR-143/145 --| *HK2*	293T, RCC	[62,63]
Membrane depolarization and Ca^2+^ influx	miR-124a2 → *Kcnj11*, *Abcc8*	MIN6	[46]
	miR-145 → *Cacna1c*	mouse smooth muscle	[64]
	miR-103 --| *Cacna1c/2d1*, *Cacnb1*	COS-7, rat neurons	[65]
	miR-328 --| *Cacna1c*, *Cacnb1*	HEK293, atrial tissues rat, mouse, dog	[66]
Exocytotic process	miR-375 --| *Mtpn*	MIN6	[67]
	miR-7a --| *Snca*, *Cspa*, *Cplx1*	MIN6, mouse islets	[68]
	miR-335 --| *Stxbp1*	INS-1 832/13	[35]
	miR-9 → *Slp4*	MIN6	[69]
	miR-29a/b/c → *Slp4*	MIN6, mouse islets	[70]
	miR-124a → *Snap25*, *Stx1a*, *Rab3A*	MIN6B1	[71]
	miR-96 → *Slp4*	MIN6B1	[71]
	miR-124a --| *Rab27A*	MIN6B1	[71]
	miR-124a, miR-96 -?-| *Noc2*	MIN6B1	[71]
	miR-34a --| *Vamp-2*	MIN6B1	[72]
	miR-29a --| Stx1a	INS-1E	[73]
Insulin gene regulation ^3^	miR-30d --| *Map4k4*	MIN6	[74]
	miR-15a --| *Ucp2*	MIN6	[75]
	miR-375 --| *PDK1*	INS-1E	[76]
	miR-24, miR-148a --| *Sox6*	mouse islets	[37]
	miR-182 --| *Bhlhe22*	mouse islets	[37]

^1^ Direct negative regulatory targeting denoted by miRNA **--|** mRNA; indirect targeting with positive effect, miRNA → mRNA; indirect targeting with negative effect, miRNA **-**?**-|** mRNA. Interactions are experimentally validated by reporter assays, and/or modulation of miRNA levels. Gene nomenclature according to HGNC guidelines, e.g., human gene: *MTPN*, ortholog rodent gene: *Mtpn* [77]*.* In some studies, rodent cell lines have been used to validate miRNA targeting of human gene 3'UTR in plasmid vectors. ^2^ Included also are studies on non-beta cell model systems where miRNA-dependent regulation of known components of GSIS has been demonstrated. ^3^ Insulin gene regulation is not a process of GSIS *per se* but an important factor in determining the amount of insulin available for subsequent release.

While many mitochondrial genes are directly implicated in beta cell functions, few studies have shown their direct regulation by miRNAs. Interestingly, in breast cancer cells and renal cell carcinoma, hexokinase-2 (HK2) has been shown to be directly regulated by miR-143/145 [62,63]. In the beta cell, the major isoform present is hexokinase-4 or glucokinase which catalyzes the phosphorylation of glucose to glucose-6-phosphate. Glucokinase is a special hexokinase isoform in the beta cells activated at higher glucose concentration than other hexokinases. It is therefore thought to be a rate-limiting factor in glucose-stimulated insulin secretion and the presence of other isoforms of hexokinase would cause hypoglycemia due to their lower K_m_ for glucose [61]. Could it be that the other hexokinase isoforms are selectively repressed in the beta cell via miRNA-mediated mechanisms?

### 3.2. Ion-Channels Controlled by miRNAs

Pancreatic beta cells are electrically active and respond to glucose stimulation with plasma membrane depolarization and the formation of bursts of action potentials. Ion channels are pore-forming proteins, present in the membranes of the beta cells and crucial for the action potential firing by permeating the flow of ions across the cell membrane. In shaping the electrical activity of pancreatic beta cells’ ATP-sensitive K^+^ channels, voltage-dependent Na^+^ channels, voltage-dependent Ca^2+^ channels and voltage or Ca^2+^-regulated K^+^ channels are essential [78].

In MIN6 beta cells, miR-124a2 has been suggested to regulate the ATP-sensitive K^+^ channel subunits kir6.2 (*KCNJ11*) and SUR1 (*ABCC8*) through the transcription factor *Foxa2* [46]. There are as of yet not many studies indicating ion channels to be direct targets of miRNAs in beta cells; however, there are studies demonstrating ion channels targeted by miRNAs in other tissues. Diabetes is associated with underlying risk for heart diseases: miR-301 was found to downregulate a voltage-dependent potassium channel (Kv4.2) in the heart of the diabetic db/db mouse model [79]. For vascular smooth muscle cells, miR-145 plays an important role in regulation of L-type calcium channel expression [64]. MiR-103 [65] and miR-328 [66] have been demonstrated to target L-type calcium channels in brain and heart, respectively. Our own preliminary results suggests that miR-375 targets subunits of the voltage-dependent Na^+^ channel, and bioinformatics analysis indicate that the target subunits differ between species [80].

### 3.3. Control of Exocytosis by miRNAs

The biosynthesis of insulin is a tightly regulated process controlled by transcription factors, as well as miRNAs. In short, miR-30d, miR-15a, miR-375, miR-24, miR-148a and miR-182 are suggested to control genes involved in insulin gene expression (Table 1). For more details regarding miRNA regulation of insulin biosynthesis, we refer to the original articles [37,74,75,76]. After being synthesized, insulin is stored in large dense-core vesicles (LDCVs) and released through Ca^2+^-dependent exocytosis.

The exocytotic process consists of several steps and involves a plethora of proteins [15], of which many are controlled by miRNAs. The first miRNA to be identified as a regulator of insulin release was miR-375 [67]. Overexpression of miR-375 in mouse beta cells reduces exocytosis evoked by cell membrane depolarizations. The effect of miR-375 on exocytosis was later verified in beta cells from the miR-375 KO mouse that has an increased depolarization-induced exocytotic response [48]. It is not yet fully established through which mechanism miR-375 regulates beta cell exocytosis, though myotrophin is identified as one possible target [67].

Fusion of the LDCVs with the plasma membrane is aided by many exocytotic proteins including the plasma-membrane SNARE-proteins; syntaxin 1, SNAP-25 and the vesicular protein VAMP-2. Recently it was shown that syntaxin 1A is targeted by miR-29a [73]. The same group has previously shown that suppression of miR-29a in INS-1E cells increases glucose-induced insulin secretion while overexpression of the miRNA has the opposite effect [81]. SNAP-25 is directly regulated by miR-153 in zebrafish [82]. In pancreatic beta cells, the direct control of SNAP-25 by miRNAs has not yet been established. However, an indirect regulation of SNAP-25 by miR-124a has been reported in MIN6B1 cells [71]. In this study, mir-124a increased the levels of SNAP-25, as well as rab 3A and synapsin 1A, via an indirect manner through unknown mechanisms.

VAMP-2 is reported to be under the direct control of miR-34a [72], and overexpression of miR-34a decreases glucose-stimulated insulin secretion in MIN6B1 cells, indicating also miR-34a as a regulator of beta cell exocytosis. It is essential for exocytosis that the SNARE complex is formed properly. Recently, miR-7 was identified as a regulator of cysteine-string protein-alpha (*cspa*) and alpha-synuclein (*snca*), proteins known to chaperone the assembly of the SNARE complex [68].

Apart from the major SNARE proteins, exocytosis of insulin containing LDCVs is aided and controlled by several other proteins. Munc-18 (or stxbp1) is a protein known to bind syntaxin 1 as well as the granular protein granuphilin [22]. In a study conducted on the non-obese T2D model, the Goto Kakizaki rat (GK rat), we detected several miRNAs that were differentially regulated compared to control Wistar rats. Of these, miR-335 was verified as a direct regulator of munc-18 [35].

The negative regulator of insulin exocytosis granuphilin has been reported to be under the indirect regulation of miR-9 [69], miR-29 a/b/c [70] and miR-96 [71]. With regards to miR-9 and miR-29 a/b/c, they act by reducing the expression of the transcription factor onecut-2. Since onecut-2 reduces transcription of the granuphilin gene a reduction in the transcription factor leads to an increase of granuphilin and reduced insulin secretion [69,70]. In the case of miR-96, the exact mechanism of its action is not yet elucidated, though this miRNA does not target onecut-2 [71]. Granuphilin is believed to interact with rab27a to regulate exocytosis of insulin-containing vesicles [83]. It has been shown that miR-124a decreases the levels of rab27a in MIN6B1 cells through direct regulatory targeting [71]. Rab27a also associates with noc2, another protein that was indicated in the latter study to be regulated by miR124a and miR-96, although in an indirect manner [71]. In conclusion, it appears that the genes involved in the exocytotic process are more regulated by miRNAs than genes within other processes of the stimulus-secretion coupling. However, this still requires further investigation.

## 4. Conclusions

Insulin secretion is a key feature in glucose metabolism, and impairment in the beta cell capacity to release enough of the hormone is central to the development of T2D. The role of miRNAs in islet functions and the pathophysiology of T2D are being widely acknowledged and, to date, the question remains to explain the exact molecular roles of miRNAs in beta cell physiology. We have in this review listed the miRNAs controlling beta cell stimulus-secretion coupling and exocytosis. It is evident that large proteins like ion-channels are not as regulated by miRNAs as smaller proteins involved in the exocytotic machinery. Putting it all together, it seems that the major pathways in beta cell physiology controlled by miRNAs are docking, priming and exocytosis. Hence, impairments in the post-transcriptional control of expression of genes involved in the late steps of insulin secretion through a change in miRNA levels might explain the reduced expression of exocytotic genes observed in islets from T2D donors. Although we are beginning to better understand how miRNAs control gene expression in beta cell physiology, there remains much to be discovered. For instance, we need more knowledge regarding the transcriptional control of miRNA levels and associated feedback loops to comprehend the complexity by which miRNAs cooperate to initiate necessary adaptation of insulin secretion in the beta cell.

In conclusion, miRNAs are vital regulators of beta cell stimulus-secretion coupling and exocytosis, and subsequently in the adaptations taking place during the development of T2D. They represent a regulation of gene expression beyond the genome that is sensitive to metabolic changes. An increased knowledge of the miRNA network and how it is regulated will be beneficial for future treatments of T2D.
